# Association of delirium with post-traumatic stress disorder: a systematic review and meta-analysis

**DOI:** 10.3389/fpsyt.2025.1654136

**Published:** 2025-09-18

**Authors:** Xia Yang, Fang Chen, Zhixia Jiang, Nan Xu, Xia Zhang, Qingqing Chen, Mingwei Cao

**Affiliations:** ^1^ School of Nursing, Zunyi Medical University, Zunyi, Guizhou, China; ^2^ Department of Intensive Care Unit, Affiliated Hospital of Zunyi Medical University, Zunyi, Guizhou, China; ^3^ College Office, Guizhou Nursing Vocational College, Guiyang, Guizhou, China

**Keywords:** delirium, post-traumatic stress disorder, systematic review, meta-analysis, ICU

## Abstract

**Background:**

Delirium, a prevalent form of acute brain dysfunction, is characterized by perceptual disturbances that may lead to the formation of delusional memories. This pathological process could subsequently elevate the risk of developing posttraumatic stress disorder (PTSD). However, the findings of previous research are inconsistent, and the association has not been systematically evaluated. Therefore, this study attempts to clarify the epidemiological relationship between delirium and PTSD as well as its clinical significance through a thorough integration of the available data, aiming to provide an evidence-based foundation for the early identification of high-risk patients and the creation of focused intervention strategies.

**Methods:**

We followed the Preferred Reporting Items for Systematic Reviews and Meta-Analyses (PRISMA) guidelines during the literature search. Comprehensive searches were conducted in PubMed, Ovid MEDLINE, APA PsycINFO, Embase, Scopus, and CINAHL, covering all relevant literature published from each database’s inception until April 3, 2025. The search strategy combined free-text terms with controlled vocabulary (MeSH/Emtree terms) related to delirium and PTSD. This systematic review was registered with PROSPERO (CRD420251031880).

**Results:**

A total of 11 articles were included in this study. Meta-analysis of unadjusted ORs revealed that patients with delirium exhibited a significantly higher risk of developing PTSD compared to non-delirium controls (OR = 3.31, 95% CI [2.21–4.97]). After adjusting for potential confounders, the pooled results based on adjusted ORs continued to indicate a significant association between delirium and increased PTSD risk (OR = 3.96, 95% CI [1.85–8.50]). Six studies explored differences in PTSD scores between delirious and non-delirious patients. Of the four studies initially reporting median values, two were excluded following skewness assessment for non-normal data distribution. The data from the remaining two studies were transformed into mean ± *SD* format for subsequent analysis. A meta-analysis of these four trials revealed that patients with delirium scored significantly higher on PTSD symptoms than those without (SMD = 0.50, 95% CI: 0.22–0.78, Z = 3.459, P<0.001).

**Conclusion:**

This meta-analysis found a significant association between PTSD and delirium.

**Systematic review registration:**

https://www.crd.york.ac.uk/PROSPERO/view/CRD420251031880, identifier CRD420251031880.

## 1 Introduction

Delirium is a syndrome of acute brain dysfunction. The American Psychiatric Association’s diagnostic criteria define delirium as the abrupt onset of acute impairment of attention, level of consciousness, and cognitive functioning over a brief period usually hours to days, caused by an underlying medical condition or drug effect. This cluster of symptoms is currently not explained by comatose states or preexisting neurocognitive deficits (1). Epidemiological studies indicate that delirium affects 14% to 24% of hospitalized patients. The number rises dramatically with the severity of the underlying illness and length of hospital stay, reaching 70% to 87% among patients in intensive care (2). Current evidence demonstrates that delirium contributes to multiple adverse clinical outcomes, including prolonged mechanical ventilation duration and ICU length of stay, increased incidence of systemic complications, and long-term cognitive impairment (3, 4).

In recent years, research has gradually revealed a potential association between delirium and PTSD. Delirium’s aberrant perceptual experiences may lead to the creation of delusional memories; this process increases the risk of developing PTSD (5). As a potential distant complication of delirium, PTSD manifests with symptoms of extreme fear. Its primary symptoms include traumatic re-experience, avoidance symptoms, negative cognitive and emotional changes, and increased alertness and responsiveness (1, 6). These symptoms severely impair people’s social functioning, drastically lower their quality of life, and place a heavy burden on both individuals and society. Therefore, it is clinically crucial to determine whether delirium is a risk factor for PTSD to create effective PTSD prevention methods.

However, current research evidence on whether delirium affects the occurrence of PTSD is significantly discrepant. According to certain cohort studies, patients with delirium have a 3–4 times greater risk of PTSD than patients without delirium (OR = 3.23, 95% CI 0.61–17.13), particularly at the 12-month follow-up time point following patient discharge (7). However, according to Su et al.’s study (8), there was no statistically significant difference in the incidence of PTSD between patients with/without delirium, nor in any of the scale assessment characteristics (hyperarousal, intrusive symptoms, and avoidance symptoms). Methodological variations, including the selection of PTSD assessment instruments, the establishment of follow-up time points, and disparate study population characteristics, could be the cause of this discrepancy. While multiple primary investigations have examined the delirium-PTSD relationship, the current literature lacks comprehensive meta-analytic quantification of this association. Therefore, there is a need to elucidate the association through systematic integration of evidence.

This study employs meta-analytic methods to quantitatively assess the delirium-PTSD association, while investigating potential heterogeneity sources through sensitivity and subgroup analyses, thereby establishing a robust evidence base for clinical decision-making.

## 2 Materials and methods

The systematic review and meta-analysis were conducted in accordance with PRISMA guidelines and have been prospectively registered in the Prospective Registry of Systematic Reviews (PROSPERO) (identifier: CRD420251031880).

### 2.1 Search strategy

This study used a systematic literature search strategy with a computerized search of six databases: PubMed, Ovid Medline, APA PsycINFO, Embase, Scopus, and CINAHL. The search covered all relevant literature published between the time each database was constructed and April 3, 2025. We developed search strategies using both subject headings and free-text terms for delirium and PTSD. Detailed search strategies are provided in **Supplementary Material**.

### 2.2 Inclusion and exclusion criteria

#### 2.2.1 Inclusion criteria

(1)Delirium diagnosis must be established using validated assessment tools (e.g., CAM or ICDSC), meet standardized diagnostic criteria as outlined in the Diagnostic and Statistical Manual of Mental Disorders (DSM) or the International Classification of Diseases (ICD), or be extracted from a case using a qualified professional’s methodology.

(2)The diagnosis of PTSD is based on clinically validated standardized assessment tools, such as diagnostic criteria based on the DSM, reference to the ICD diagnostic criteria, or reliability-tested structured clinical interview tools (e.g., CAPS-5) and symptom rating scales (e.g., PCL-5 or IES-R).

(3) Only prospective or retrospective observational analyses were included.

(4) Articles must be published in English.

(5) Articles must be of moderate or high quality, which is assessed through the Nottingham Ottawa Scale.

#### 2.2.2 Exclusion criteria

(1) Studies for which the full text was not available, the raw data were incomplete, or not reported.

(2) Non-observational studies and review articles.

(3) Oral reports, case reports, newspaper and meeting abstracts.

### 2.3 Literature selection

After using Endnote 21 document management software to remove duplicates of the literature found through the search, two researchers independently conducted a preliminary screening of the remaining literature’s titles and abstracts. Those that did not fit the inclusion criteria were immediately eliminated. The full-text material that satisfied the criteria was then examined by two researchers, and any disagreements that surfaced throughout the screening process were settled by discussion or third-party negotiation.

### 2.4 Data extraction and quality evaluation

Two researchers conducted full-text data extraction following the completion of the literature screening. The data extracted included the first author, year of publication, study locations, study population, age, sample size, gender ratio, duration of follow-up, delirium and PTSD assessment tools, and assessment details. Odds ratios (OR) with 95% confidence intervals (CI) and other effect sizes quantifying the relationship between delirium and PTSD were also extracted, using only data from the last follow-up point.

This study used the Nottingham Ottawa Scale (NOS) (9) to evaluate the quality of the included studies. The NOS evaluates studies based on three domains: research subject selection, between-group comparability, and outcome measurements. The scale rating criteria state that the total score ranges from 0 to 9, where 5–6 indicates research of moderate quality and 7–9 indicates studies of high quality. To ensure the reliability of the analysis results, only medium-quality and high-quality literature with a score of ≥5 was selected for inclusion in the final analysis of this study.

### 2.5 Data synthesis and analysis

This study describes in detail the design features, methodological quality, and results of each study through a narrative synthesis and structured tables. Depending on the type of data distribution, mean ± *SD* or median *(IQR*) was selected to report continuous variables. Dichotomous variables were reported as counts and percentages (%). During the effect size data extraction phase, we collected both adjusted and unadjusted ORs and their 95% CIs, as well as the raw data used to calculate the ORs and 95% CIs. Based on this, we used the Mantel-Haenszel method to combine and weight the adjusted ORs and unadjusted ORs separately to assess the strength of the association between exposure and outcome before and after controlling for confounding factors. If the combined OR value is greater than 1, it indicates that the risk of event occurrence in the exposed group is higher than that in the control group, suggesting that the exposure factor may be a risk factor. Conversely, if the OR value is less than 1, it suggests that the exposure factor may be a protective factor. For risk estimate measures such as OR, Ferguson (10) proposed the following interpretation criteria: OR≥2.0 is considered the recommended minimum effect size (RMPE), ≥3.0 indicates a moderate effect, and≥4.0 signifies a strong effect. However, given that the statistical properties of OR are not anchored to *r*, caution should be exercised when interpreting these values.

For articles where the raw data are reported as median(IQR), the data are first monitored for deviations from normality (11). If the normality test shows that the data are not significantly skewed, the sample mean and standard deviation can be estimated according to the best statistical methods recommended by the existing literature (12, 13). The standardized mean difference (SMD) was used as the effect size. SMD > 0 indicates that the average outcome in the exposed group is higher than that in the control group, while SMD < 0 indicates that the average outcome in the exposed group is lower than that in the control group. For between-group effect sizes, Ferguson (10) recommends the following interpretation criteria: 0.41 represents RMPE, 1.15 corresponds to a moderate effect, and 2.70 corresponds to a large effect. Heterogeneity was assessed using the Cochrane Q-test and the I²statistic. Based on conventional thresholds (14) (low heterogeneity for I²≤30%, moderate for 30% < I²≤50%, and high for I²> 50%), a fixed effects model was chosen if the Q-test p-value>0.1 and *I²*<50%; if not, a random effects model was employed.

The influence of individual articles on the total effect size was first evaluated via sensitivity analysis to investigate possible causes of heterogeneity. Then, the origins of heterogeneity were investigated using subgroup analyses depending on important variables such as the kind of study design, the instruments used for evaluation, and the length of follow-up. When ten or more articles were included, publication bias was evaluated using Egger’s test and visual inspection of funnel plots; however, because statistical tests were not always as effective when fewer than ten studies were included, quantitative bias analysis was not carried out.

## 3 Results

### 3.1 Selected research

**Figure 1:**
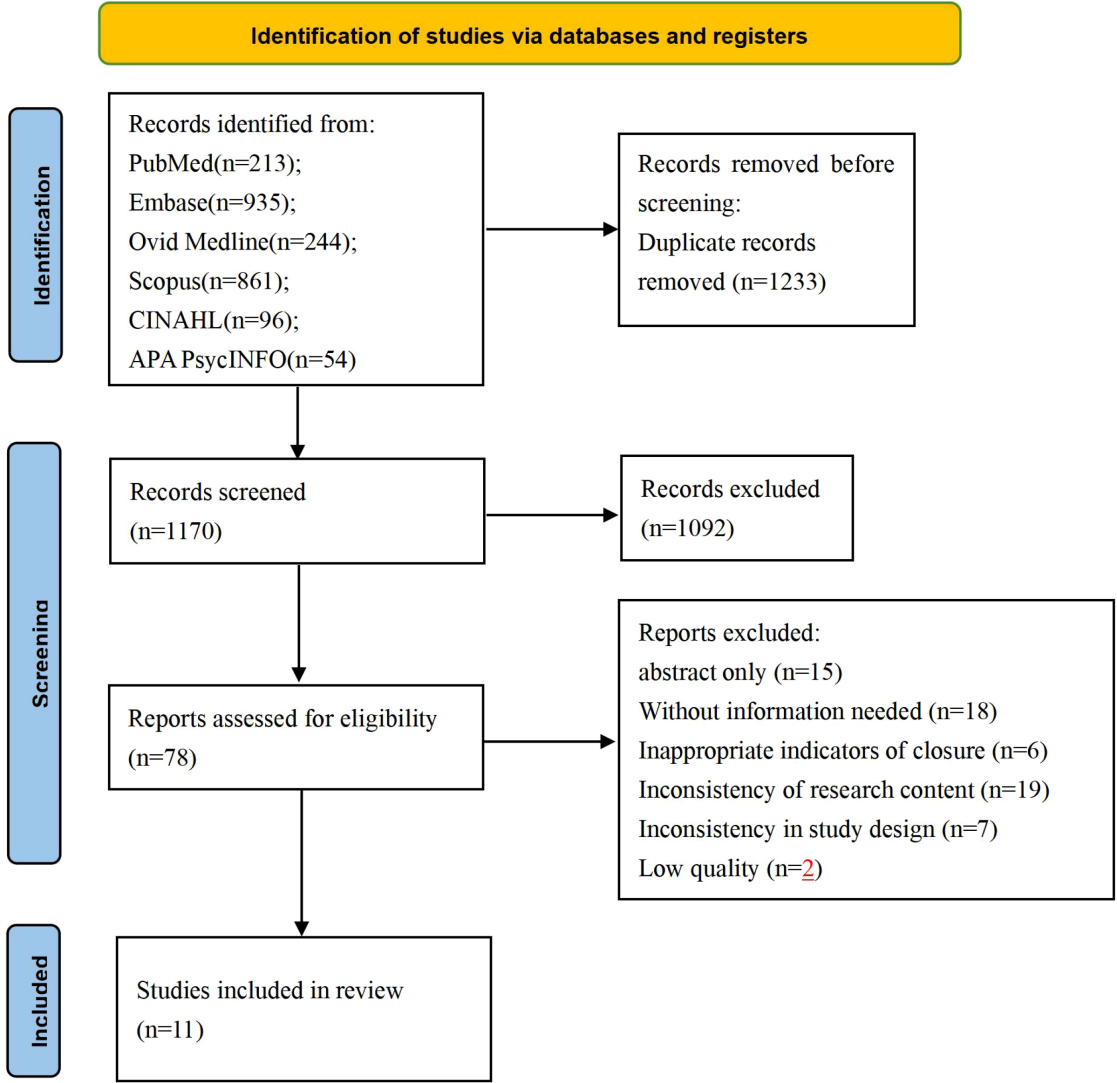
The process of literature screening.

**Figure 1** outlines the complete literature search process. The initial search resulted in a total of 2403 documents, with 1170 remaining after de-duplication. 1092 articles that did not meet the inclusion criteria were excluded by title and abstract screening. The remaining 78 articles were then assessed in full text, and 67 articles were excluded due to incompatible relevant data, study design, and study content. Ultimately, 11 articles were included in this study. The inter-rater reliability of the literature screening process was good, with a Cohen’s Kappa value of 0.76 (p < 0.001).

### 3.2 Characteristics of included studies

**Table 1** summarizes the basic characteristics of the included studies. A total of 11 articles involving 2533 study participants were included in this study. Geographically, two of the studies were from the Americas, two from Asia, one from Australia, and six from Europe. Eight studies involved intensive care unit patients, two involved geriatric populations, and one involved patients following hematopoietic cell transplant.

**Table 1:** Study characteristics.

Study year	Site	Participants				Assessment tools		Follow-up period
		Age (m ± SD)	Sample size n (ND/D)	Sex, male n(%)	Research population	Delirium	PTSD	
Basinski 2010 (15)	America	NA	52(29/23)	29 (55.8%)	HCT	DRS	PTS	1 year
Slor 2013 (16)	Netherlands	83.3 ± 5.7	53(30/23)	12 (22.6%)	Elderly Hip Fractures	CAM	PTSS-10	3 months
Drews 2015 (17)	German	NA	559(482/77)	304 (54.4%)	Elderly	CAM	PTSS-14	3 months
Svenningsen 2015 (18)	Denmark	62 (40–78)^d^	299(138/161)	166 (56%)	ICU	CAM-ICU	HTQ	6 months
Wolters 2016 (19)	Netherlands	57.69 ± 16.33^a^	567(270/297)	334 (59%)	ICU	CAM-ICU	IES-15	1 year
Battle 2017 (20)	Britain	64 (53–73)^b^	198(163/35)	92 (46%)	ICU	Medical record acquisition	UK-PTSS-14	3 months
Bulic 2020 (21)	Australia	60 ± 16	103(66/37)	53 (52%)	ICU	CAM-ICU	IES-R	1 year
Miyamoto 2021 (7)	Japan	72 (61–81)^b^	204(143/61)	121 (59%)	ICU	CAM-ICU	IES-R	1 year
Rocha 2023 (22)	Brazil	53 ± 17	65(56/9)	40 (62%)	ICU	CAM-ICU	IES-6	4 months
Friberg 2024 (23)	Norway	61 (50–70)^b^	273(242/31)	175 (64.1%)	ICU	CAM-ICU	IES-R	3 months
Su 2024 (8)	China	52 (45–61)^b^	160(80/80)	96 (60.0%)	ICU	CAM-ICU	IES-R	3 months

NA, Unavailable; ND, No delirium; D, Delirium; HCT, Hematopoietic Cell Transplant; DRS, the Delirium Rating Scale; PTS, The Post Traumatic Stress scale; CAM, Confusion Assessment Method; PTSS-10, The Post-Traumatic Stress Syndrome Scale-10; CAM-ICU, The Confusion Assessment Method for the ICU; HTQ, The Harvard Trauma Questionnaire; IES-15, the Impact of Event Scale 15 item version; UK-PTSS-14, the UK-Post-Traumatic Stress Syndrome 14-Questions Inventory; IES-R, Impact of events scale-revised; IES-6, the Impact of Event Scale-6.

^a^represents the combined calculation result.

^b^represents median (interquartile spacing).

^d^represents median (10; 90 percentile).

Four principal methods were employed for delirium diagnosis across studies. While multiple instruments were available, the Confusion Assessment Method (CAM; n=2) and its intensive care-adapted version (CAM-ICU; n=7) emerged as the most frequently utilized tools. For PTSD assessment, five validated scales were implemented: the PTSD Symptom Scale (PTS; n=1), Harvard Trauma Questionnaire (HTQ; n=1), PTSD Symptom Scale series (PTSS; n=3), and Impact of Event Scale series (IES; n=6). Notably, four studies adopted the IES-R (Impact of Event Scale-Revised), which quantifies symptom severity on a 0–88 continuum, with higher scores indicating greater distress. However, diagnostic thresholds varied significantly across studies: Bulic 2020 >37, Miyamoto (7) used ≥25, Friberg (23) ≥33, and Su (8) ≥35 points. Most studies evaluated PTSD symptoms at either 3-month (n=5) or 12-month (n=4) post-discharge intervals. The results of the included studies are presented in the **Supplementary Material**.

### 3.3 Quality assessments

As shown in **Table 2**, the methodological quality of all included studies was moderate to high. The absolute consistency intraclass correlation coefficient (ICC) was calculated using a two-way random effects model to assess the consistency between two reviewers on the total score of the NOS. The analysis results showed good inter-rater consistency, with a single measurement ICC value of 0.82 (95% CI: 0.48–0.95), and this was statistically significant (p < 0.001). However, the study also had some methodological limitations. Regarding participant selection (Q4), most articles (n=9) did not specify whether patients with mental disorders were included or whether patients with PTSD at baseline were excluded. Eight studies were deemed to have insufficient follow-up because they either had a dropout rate exceeding 20% or did not compare key characteristics between dropouts and non-dropouts.

**Table 2:** NOS scores for included studies.

Study year	Q1	Q2	Q3	Q4	Q5	Q6	Q7	Q8	Q9	Scores
Basinski 2010 (15)	Y	Y	Y	U	Y	Y	Y	Y	N	7
Slor 2013 (16)	Y	Y	Y	U	Y	Y	Y	Y	N	7
Drews 2015 (17)	Y	Y	Y	U	Y	Y	Y	Y	N	7
Svenningsen 2015 (18)	Y	Y	Y	Y	Y	N	Y	Y	Y	8
Wolters 2016 (19)	Y	Y	Y	U	Y	Y	Y	Y	N	7
Battle 2017 (20)	Y	Y	Y	N	Y	N	Y	Y	U	6
Bulic 2020 (21)	Y	Y	Y	Y	Y	N	Y	Y	N	7
Miyamoto 2021 (7)	Y	Y	Y	U	Y	Y	Y	Y	N	7
Rocha 2023 (22)	Y	Y	Y	U	Y	N	Y	Y	N	6
Friberg 2024 (23)	Y	Y	Y	U	Y	N	Y	Y	Y	7
Su 2024 (8)	Y	Y	Y	U	Y	Y	Y	Y	Y	8

Q1 Representativeness of the exposed cohort.

Q2 Selection of the non-exposed cohort.

Q3 Ascertainment of exposure.

Q4 Demonstration that outcome of interest was not present at start of study.

Q5 Study controls for _(select the most important factor).

Q6 Study controls for any additional factor.

Q7 Assessment of outcome.

Q8 Was follow-up long enough for outcomes to occur.

Q9 Adequacy of follow-up of cohorts.

Y, Yes; N, No; U, Unclear.

### 3.4 Meta-analysis

#### 3.4.1 Meta-analysis related to PTSD based on unadjusted OR values

**Figure 2:**
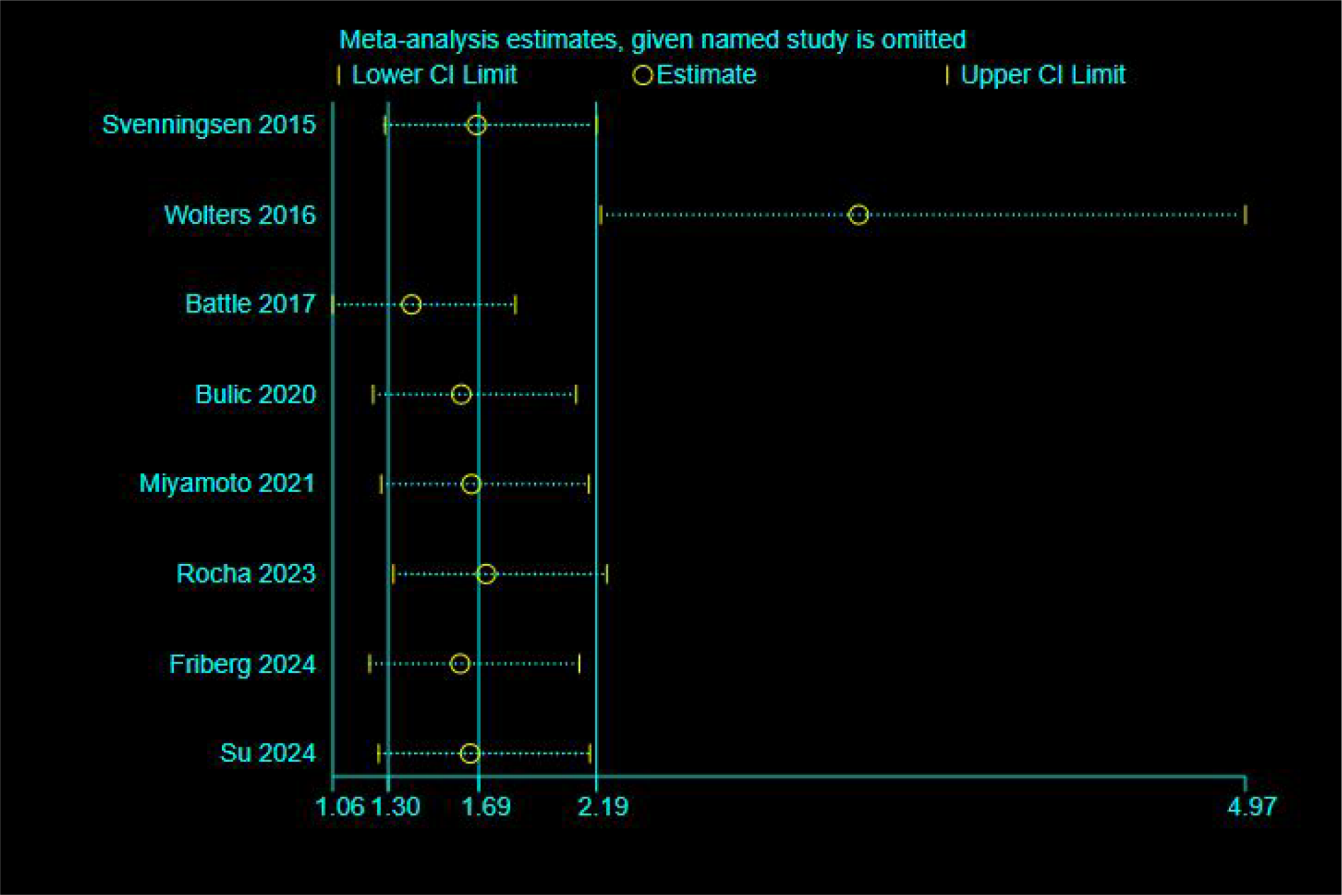
Sensitivity analysis (unadjusted odds ratio).

**Figure 3:**
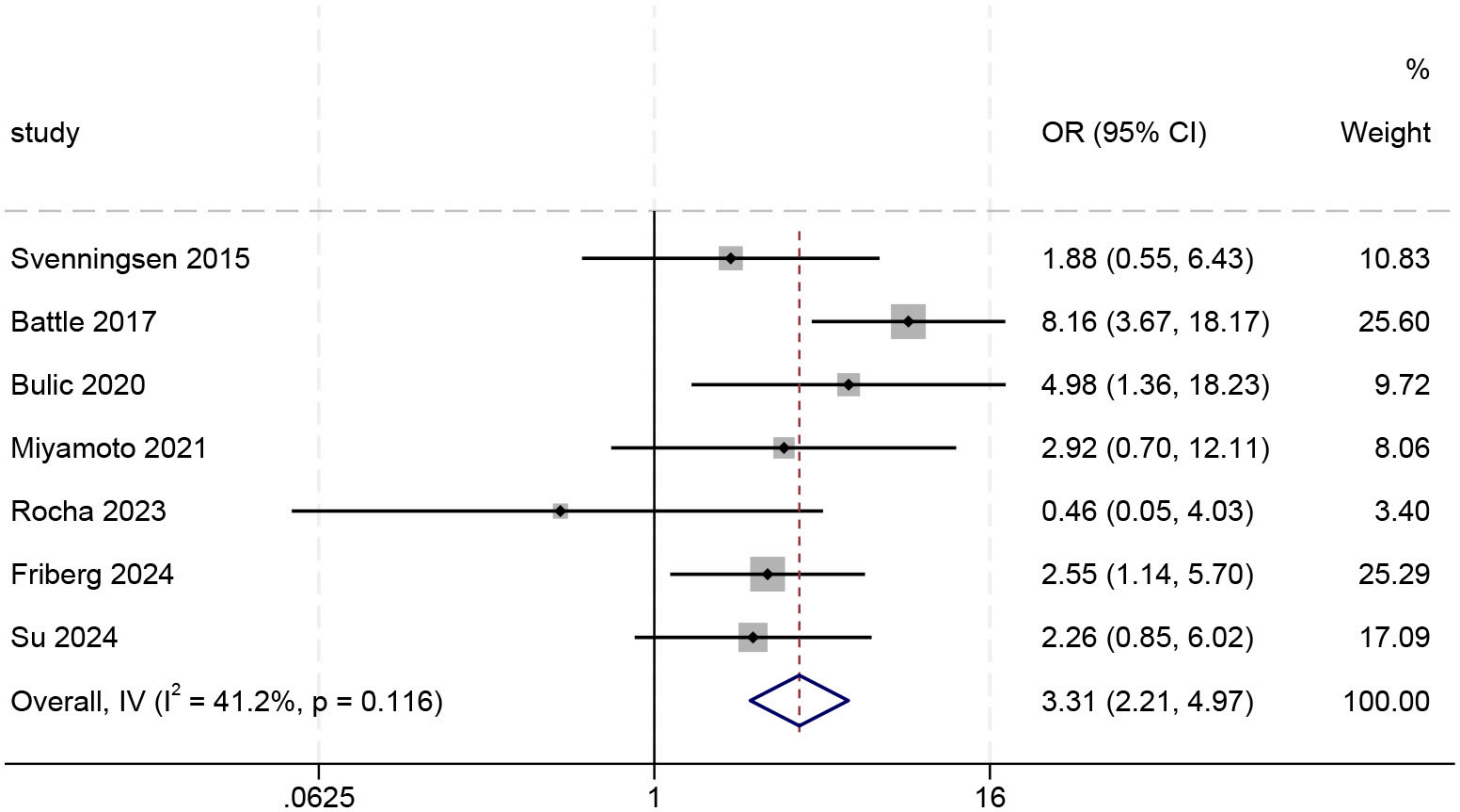
Forest plot of unadjusted odds ratios (n=7).

Meta-analysis of ORs from eight included studies revealed a pooled OR of 1.69 (95% CI: 1.30–2.19, p < 0.001), with high heterogeneity (I²= 75.3%, p < 0.001). (See **Supplementary Materials** for the forest plot.) To assess the robustness of the pooled results, we conducted a sensitivity analysis using the “leave-one-out” method (see **Figure 2**). The results suggest that the study by Wolters et al. (19) had a substantial impact on both the pooled effect size and the observed heterogeneity. Following its exclusion, heterogeneity decreased to a moderate level (I²= 41.2%, p > 0.1), falling below the conventional threshold of 50%. Given that this study was a pronounced outlier, it was excluded from the final analysis, and the results from the remaining seven studies were taken as the basis. Analysis of the seven studies retained after exclusion yielded a pooled OR of 3.31 (95% CI: 2.21–4.97), which was statistically significant (Z = 5.802, p < 0.001). These results indicate that patients with delirium have a significantly higher risk of developing PTSD compared to those without delirium (see **Figure 3**). Less than 10 studies were included in all meta-analyses, which would not be enough to test power; hence, publication bias was not evaluated (24).

**Figure 4:**
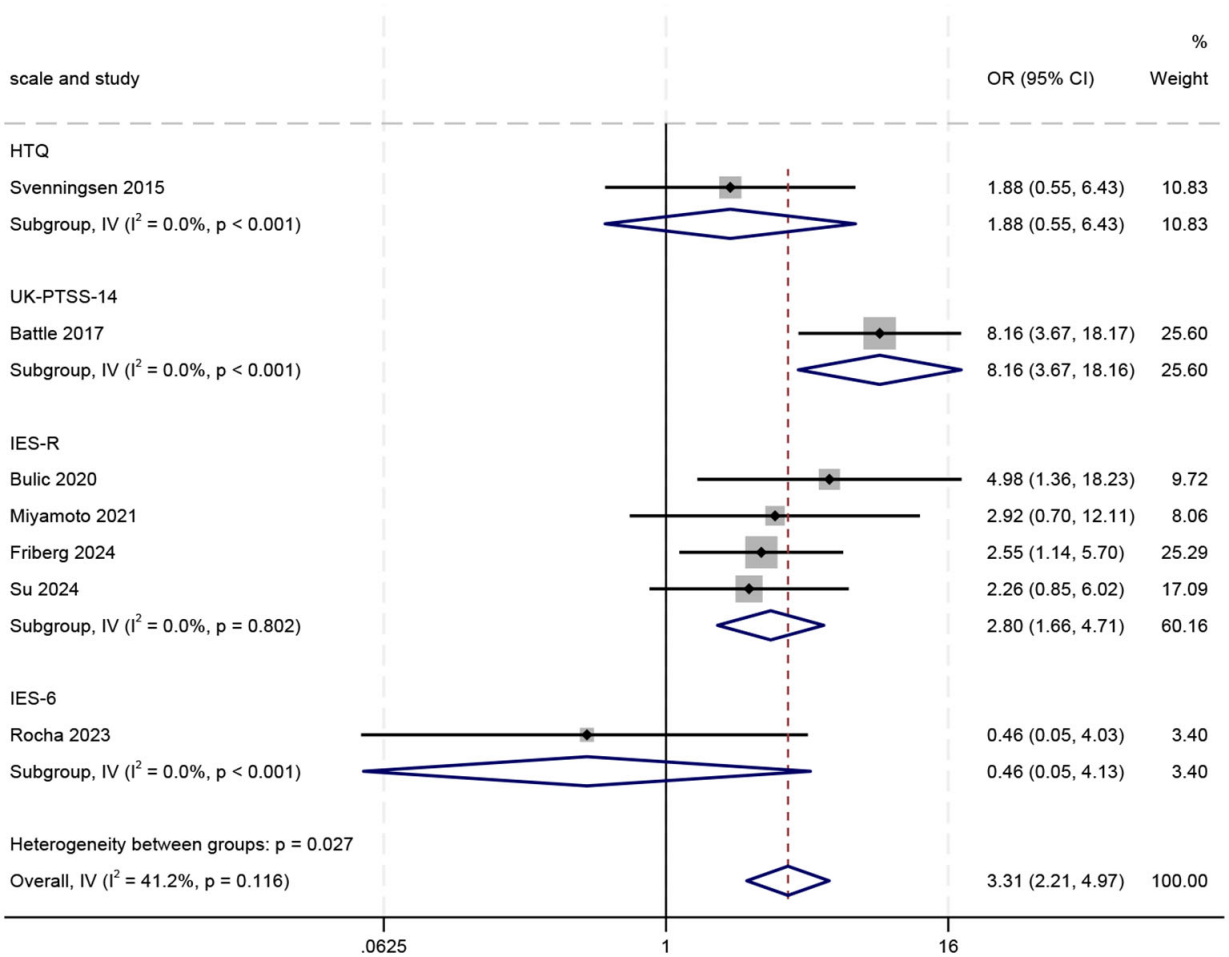
Forest plot of subgroup analyses grouped by PTSD assessment tool.

Subgroup analyses based on different PTSD assessment tools revealed statistically significant differences between groups (p = 0.027), suggesting that assessment tools may be a major source of heterogeneity across studies (see **Figure 4**). However, the UK-PTSS-14, HTQ, and IES-6 subgroups each contained only a single study, and their results were associated with considerable uncertainty(wide confidence intervals encompassing non-significant values). Therefore, no reliable conclusions can be drawn regarding the associations under these specific assessment tools. In contrast, the IES-R subgroup provided more robust evidence. This subgroup comprised four studies and exhibited no heterogeneity (I² = 0.0%, p > 0.1). The pooled analysis demonstrated a significant association between delirium and an increased risk of PTSD (OR = 2.80, 95% CI: 1.66–4.71; z = 3.862, p < 0.001), indicating that the risk of developing PTSD was approximately 2.8 times higher in patients with delirium than in those without, among studies using the IES-R scale.

#### 3.4.2 Meta-analysis related to PTSD based on adjusted OR values

**Figure 5:**
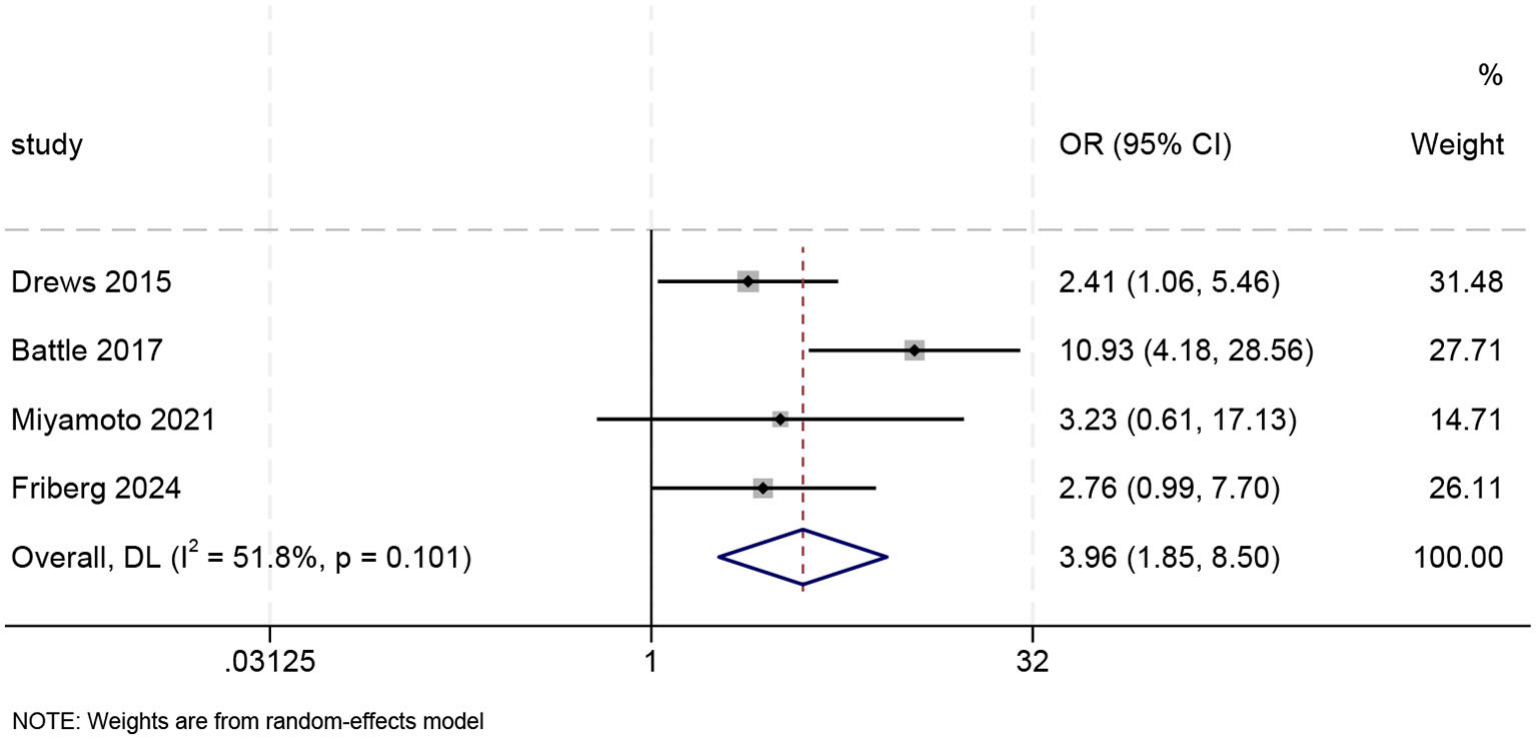
Forest plot of adjusted odds ratios (n=4).

Meta-analysis of four studies reporting adjusted ORs was performed using a random-effects model. The pooled analysis yielded a combined adjusted OR of 3.96 (95% CI: 1.85–8.50), which was statistically significant (p < 0.001). The results indicate that, after adjusting for potential confounding factors, patients with delirium had a 3.96-fold increased risk of developing PTSD compared to those without delirium (see **Figure 5**). The heterogeneity reached a moderate level (I² = 51.8%), so we employed a random-effects model for pooling. To assess the robustness of the pooled results, we conducted a sensitivity analysis (see **Supplementary Materials**). The results indicate that sequentially excluding individual studies did not alter the direction of the pooled effect estimate, and the 95% confidence intervals largely overlapped with those of the original analysis. This suggests that the primary findings of the study are robust and reliable.

#### 3.4.3 Meta-analysis related to PTSD based on continuous variables

**Figure 6:**
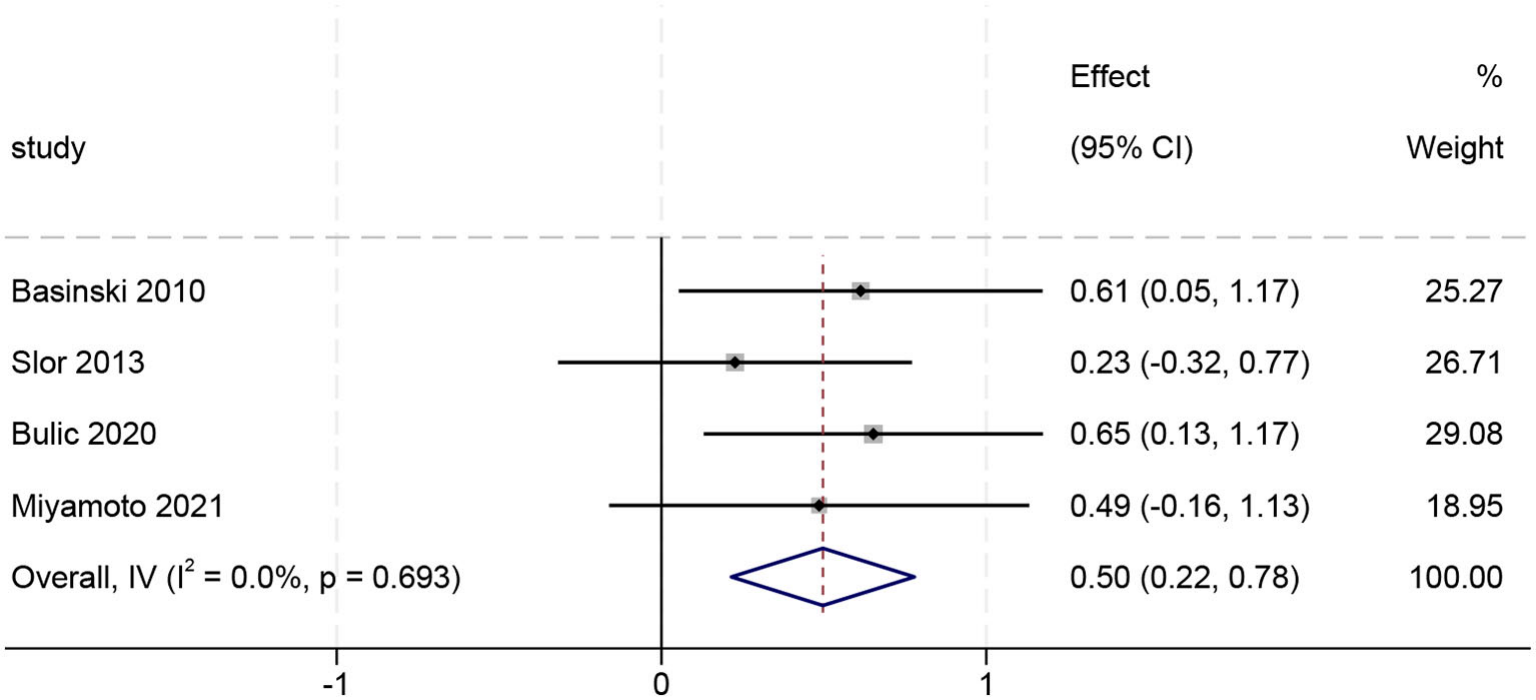
Forest plot of continuous variables (n=4).

Among the six studies comparing PTSD scores between delirium and non-delirium groups, four reported median values. Normality tests (skewness assessment) identified non-parametric distributions in two datasets, resulting in the exclusion of two studies, Svenningsen 2015 and Su 2024. The final analysis included four studies, whose data were converted for pooling (see **Supplementary Materials** for details of the converted values). Using a fixed-effects model, the pooled analysis revealed no between-study heterogeneity (I² = 0%, p>0.1). Sensitivity analysis (leave-one-out method) results showed that the combined results were robust, with no significant outliers identified (see **Supplementary Materials** for figures). As shown in **Figure 6**, delirium patients exhibited significantly higher PTSD symptom scores than non-delirium patients (SMD = 0.50, 95% CI: 0.22–0.78; Z = 3.459, p<0.001).

## 4 Discussions

To the best of our knowledge, this study constitutes this is the first systematic review and meta-analysis to investigate the prevalence of PTSD among patients with delirium. The meta-analysis found a significant association between delirium and PTSD. A meta-analysis of unadjusted ORs demonstrated that patients with delirium exhibited a significantly higher risk of developing PTSD compared to non-delirium controls (OR = 3.31, 95% CI [2.21–4.97]). After adjusting for potential confounders, pooled results based on adjusted ORs continued to indicate a significant association between delirium and increased PTSD risk (OR = 3.96, 95% CI [1.85–8.50]). The continuous variable meta-analysis revealed significantly greater PTSD symptom severity in delirium patients compared to controls, with a standardized mean difference of 0.50 (SMD = 0.50, 95% CI [0.22–0.78]).

To ensure the reliability of the pooled results, we excluded Wolters 2016 from the sensitivity analysis due to its disproportionate influence on heterogeneity and the pooled effect size. After excluding this study, heterogeneity decreased significantly (I²from 75.3% to 41.2%), while the direction and statistical significance of the pooled effect size remained unchanged. However, the preliminary assessment did not identify any significant differences between this study and others in terms of patient population, intervention measures, or outcome measurements. Based on this, we speculate that this anomalous result may be due to unmeasured or unreported confounding factors. Alternatively, it could be attributable to chance effects, such as sampling error, thus introducing bias that does not reflect true inter-study differences.

The precise mechanism of action between delirium and PTSD remains unclear. However, existing evidence indicates that the two conditions may be related through multilevel pathophysiological pathways, primarily involving limbic system dysfunction, hypothalamic-pituitary-adrenal (HPA) axis dysregulation, and disruption of neurotransmitters. First, delirium is associated with dysregulation of neurotransmitter systems, including dopamine, acetylcholine, and γ-aminobutyric acid (GABA) (25). Such neurochemical disturbances lead to neuronal instability. Second, both conditions are profoundly linked to dysfunction of the HPA axis. Patients who exhibit delirium symptoms frequently also have hyperactivation of the HPA axis, which results in abnormally high cortisol levels (26, 27). PTSD is characterized by hypocortisolism, manifesting as a distinct ‘low cortisol-high reactivity’ neuroendocrine profile (28, 29). This phenomenon may result from impaired negative feedback regulation of the HPA axis. This impairment follows glucocorticoid depletion induced by chronic stress and leads to a lowered response threshold, consequently causing hypersensitization to stress stimuli (2, 30). Lastly, both have the neurobiological characteristics of the amygdala’s heightened reactivity and the hippocampus’s sensitivity. These abnormalities in limbic system functioning can result in maladaptive consolidation of traumatic memories and poor emotion control (2, 25, 31).

This meta-analysis involved four different PTSD assessment tools. Subgroup analysis based on the assessment tool revealed statistically significant between-group differences (p = 0.027), suggesting that the type of instrument used is a potential source of heterogeneity. However, except for the IES-R subscale, which combined multiple studies, each of the remaining subgroups included only one study, resulting in considerable uncertainty in their findings. Therefore, conclusions drawn from this subgroup analysis should be interpreted with caution and require further validation in future research. The IES-R is the most commonly used tool for assessing PTSD in this study, and this scale is widely applied across diverse populations who have experienced acute traumatic events (32). A prospective study by Schütte et al. (33) provides evidence that the IES-R is a suitable scale for predicting the development of stress symptoms and PTSD following acute psychological trauma. However, differences in study design may lead to variations in the implementation of assessment tools, such as differences in scale structures or cutoff scores (34). For example, the studies included in our analysis used different cutoff values for the IES-R (e.g., 25, 33, and 35), a practice that could introduce fluctuations in PTSD scoring outcomes. Although assessment tools of different types vary in format and structure, they consistently capture the core symptoms of PTSD (e.g., post-traumatic experiences, avoidance behaviors, and negative cognitive changes) (34).

The included studies were centered on the ICU patient, according to the findings of this systematic review. Patients in intensive care units have distinct clinical traits. ICU patients may be particularly prone to delirium due to the severity of their illness, multimorbidity burden, mechanical ventilation, the use of sedatives and analgesics, and the psychologically stressful environment of the intensive care unit (35, 36). Research data show that the incidence of ICU delirium is as high as 70%-87%, which is significantly greater than the rate for patients in normal wards (2). Second, because of potentially traumatic elements like the severity of the disease, invasive procedures, anesthesia, worries about the disease’s prognosis, dread of dying, or witnessing another patient die, intensive care unit patients are frequently more likely to experience PTSD during treatment (37). According to the evidence currently available, PTSD can occur up to 3-4.3 times more frequently in post-ICU treatment survivors than in the general hospitalized population (38, 39). This observation prompts inquiry into whether the elevated prevalence of PTSD among ICU patients is mediated by delirium as an intermediary variable, or stems from the independent direct effects attributable to the ICU environment itself. Future prospective multicenter cohort studies can control for ICU variables to examine the relationship between delirium and PTSD.

We must recognize several limitations of our meta-analysis. Methodologically, our exclusive use of ORs and SMD as effect measures ensured analytical consistency but concomitantly constrained the pool of eligible studies for meta-analysis, potentially compromising the external validity and generalizability of our findings. Second, although this study pooled both unadjusted and adjusted ORs to mitigate confounding bias, only four studies provided adjusted estimates. Consequently, the reliability and generalizability of the findings are constrained. The interpretation of these results should therefore be cautious, and future studies should report multivariate-adjusted effect sizes to strengthen the evidence for this association. Third, this study only included academic articles published in English, which may result in language bias. Finally, even though this study identified a significant association between delirium and PTSD, the current evidence base is still lacking because only 11 studies were included, which may have an impact on the validity of the results.

## 5 Conclusion

Our meta-analysis reveals a potential association between delirium and PTSD, with the available evidence suggesting that delirium may be a risk factor for subsequent PTSD development. However, given the methodological limitations of the included studies, these findings should be interpreted with caution and require further validation through rigorously designed, high-quality studies with standardized methodologies. Given the severe impact of PTSD on patients’ long-term quality of life and social functioning, the inclusion of delirium prevention and management in the mental health intervention system has important public health implications. On the other hand, the neurobiological mechanisms via which delirium results in PTSD remain poorly understood. Future research could examine how trauma memories and delirium-related neuroinflammatory responses interact, as well as the particular correlations between various delirium subtypes (such as hyperactive/hypoactive/mixed) and the symptoms of post-traumatic stress disorder.

## Data Availability

The original contributions presented in the study are included in the article/**Supplementary Material**. Further inquiries can be directed to the corresponding author.
