# Supplementary material for: Association of delirium with post-traumatic stress disorder: a systematic review and meta-analysis
Source: Front Psychiatry. 2025 Sep 18;16:1654136. doi: 10.3389/fpsyt.2025.1654136 (PMC12488650; doi:10.3389/fpsyt.2025.1654136)

Supplementary Material

Table 1 Search strategy in Search strategies for each database

Table 2 Median-to-mean conversion

Figure 1 Forest plot of unadjusted odds ratios (n=8)

Figure 2 Sensitivity analysis (adjusted odds ratio)

Figure 3 Sensitivity analysis (continuous variables)

Table 1 Search strategy in Search strategies for each database

Pubmed	#1 "delirium" OR "Delirium of Mixed Origin" OR "Mixed Origin Delirium" OR "Mixed Origin Deliriums" OR "Subacute Delirium" OR "Deliriums, Subacute" OR "Delirium, Subacute" OR "Subacute Deliriums"<br><br>#2 "Stress Disorders, Post-Traumatic" OR "Post-Traumatic Stress Disorder" OR "Stress Disorder, Post-Traumatic" OR "Post Traumatic Stress Disorder" OR "Neuroses, Post-Traumatic" OR "Neuroses, Post Traumatic" OR "Post-Traumatic Neuroses" OR "PTSD" OR "Stress Disorder, Post Traumatic" OR "Post-Traumatic Stress Disorders" OR "Post Traumatic Stress Disorders" OR "Posttraumatic Stress Disorders" OR "Posttraumatic Stress Disorder" OR "Stress Disorder, Posttraumatic" OR "Stress Disorders, Posttraumatic" OR "Neuroses, Posttraumatic" OR "Posttraumatic Neuroses" OR "Acute Post-Traumatic Stress Disorder" OR "Acute Post Traumatic Stress Disorder" OR "Chronic Post-Traumatic Stress Disorder" OR "Chronic Post Traumatic Stress Disorder" OR "Delayed Onset Post-Traumatic Stress Disorder" OR "Delayed Onset Post Traumatic Stress Disorder" OR "Moral Injury" OR "Injury, Moral"<br><br>#3 #1 AND #2
Ovid Medline	#1 Delirium/<br><br>#2 "Delirium or delirium of mixed origin or mixed originmixed origin deliriums or<br><br>subacutedeliriums, subacute or delirium, subacute or subacute deliriums).ab.ti.kw.<br><br>#3 Stress Disorders, Post-Traumatic/<br><br>#4 "stress disorders, post-traumatic or post-traumatic stress disorder or stress disorder, post-traumatic or post traumatic stress disorder or neuroses, post-traumatic or neuroses, posttraumatic or post-traumatic neuroses or ptsd or stress disorder, post traumatic or posttraumatic stress disorders or posttraumatic stress disorders or posttraumatic stressdisorder or stress disorder, posttraumatic or stress disorders, posttraumatic or neuroses,posttraumatic or posttraumatic neuroses or acute post-traumatic stress disorder or acute post traumatic stress disorder or chronic post-traumatic stress disorder or chronic posttraumatic stress disorder or delayed onset post-traumatic stress disorder or delayed onsetpost traumatic stress disorder or moral injury or injury, moral or moral injuries).ab,ti.kw.<br><br>#5 #1 or #2<br><br>#6 #3 or #4<br><br>#7 #5 and #6
APA	(SU"delirium" OR TI("Delirium of Mixed Origin" OR "Mixed Origin Delirium" OR

PsycINFO	"Mixed Origin Deliriums" OR "Subacute Delirium" OR "Deliriums, Subacute" OR "Delirium, Subacute" OR "Subacute Deliriums") OR AB("Delirium of Mixed Origin" OR "Mixed Origin Delirium" OR "Mixed Origin Deliriums" OR "Subacute Delirium" OR "Deliriums, Subacute" OR "Delirium, Subacute" OR "Subacute Deliriums")) AND (SU"Stress Disorders, Post-Traumatic" OR TI("Post-Traumatic Stress Disorder" OR "Stress Disorder, Post-Traumatic" OR "Post Traumatic Stress Disorder"OR "Neuroses, Post-Traumatic" OR "Neuroses, Post Traumatic" OR "Post-Traumatic Neuroses" OR "PTSD" OR "Stress Disorder, Post Traumatic" OR "Post-Traumatic Stress Disorders" OR "Post Traumatic Stress Disorders" OR "Posttraumatic Stress Disorders" OR "Posttraumatic Stress Disorder" OR "Stress Disorder, Posttraumatic" OR "Stress Disorders, Posttraumatic" OR "Neuroses, Posttraumatic" OR "Posttraumatic Neuroses" OR "Acute Post-Traumatic Stress Disorder" OR "Acute Post Traumatic Stress Disorder" OR "Chronic Post-Traumatic Stress Disorder" OR "Chronic Post Traumatic Stress Disorder" OR "Delayed Onset Post-Traumatic Stress Disorder" OR "Delayed Onset Post Traumatic Stress Disorder" OR "Moral Injury" OR "Injury, Moral" OR "Moral Injuries") OR AB("Post-Traumatic Stress Disorder" OR "Stress Disorder, Post-Traumatic" OR "Post Traumatic Stress Disorder"OR "Neuroses, Post-Traumatic" OR "Neuroses, Post Traumatic" OR "Post-Traumatic Neuroses" OR "PTSD" OR "Stress Disorder, Post Traumatic" OR "Post-Traumatic Stress Disorders" OR "Post Traumatic Stress Disorders" OR "Posttraumatic Stress Disorders" OR "Posttraumatic Stress Disorder" OR "Stress Disorder, Posttraumatic" OR "Stress Disorders, Posttraumatic" OR "Neuroses, Posttraumatic" OR "Posttraumatic Neuroses" OR "Acute Post-Traumatic Stress Disorder" OR "Acute Post Traumatic Stress Disorder" OR "Chronic Post-Traumatic Stress Disorder" OR "Chronic Post Traumatic Stress Disorder" OR "Delayed Onset Post-Traumatic Stress Disorder" OR "Delayed Onset Post Traumatic Stress Disorder" OR "Moral Injury" OR "Injury, Moral" OR "Moral Injuries"))
Embase	#1 "delirium"/exp<br><br>#2 "acute delirium":ti,ab,kw OR "chronic delirium":ti,ab,kw OR "delier":ti,ab,kw OR "delire":ti,ab,kw OR "deliria":ti,ab,kw OR "delirious manifestation":ti,ab,kw OR "delirious state":ti,ab,kw OR "delirious syndrome":ti,ab,kw OR "delirium acutum":ti,ab,kw OR "delirium":ti,ab,kw<br><br>#3 "posttraumatlc stress disorder"/exp<br><br>#4 "post-traumatic stress":ti,ab,kw OR "post-traumatic stress disorder":ti,ab,kw OR "post-traumatic stress disorders":ti,ab,kw OR "posttraumatic neurosis":ti,ab,kw OR "posttraumatic psychic syndrome":ti.ab.kw OR "posttraumatic psychosis":ti,ab,kw OR "posttraumatic stress":ti,ab,kw OR "posttraumatic syndrome":ti,ab,kw OR "psychosis, posttraumatic":ti,ab,kw OR "ptsd":ti,ab,kw OR "ptsd"posttraumatic stress disorder)":ti.ab,kw OR "stress disorders, post-traumatic":ti,ab,kw OR "stress disorders, traumatic":ti,ab,kw OR "stress, posttraumatic":ti,ab,kw OR "trauma and stressor related disorders":ti,ab,kw OR "traumatic stress":ti,ab,kw OR "traumatic stress disorder":ti,ab,kw OR "traumatic stress disorders":ti,ab,kw OR "posttraumatic stress

	disorder":ti,ab,kw<br><br>#5 #1 OR #2<br><br>#6 #3 OR #4<br><br>#7 #5 AND #6
Scopus	TITLE-ABS-KEY("delirium" OR "Delirium of Mixed Origin" OR "Mixed origin Delirium" OR "Mixed Origin Deliriums" OR"Subacute Delirium" OR "Deliriums, subacute" OR "subacute Delirium" OR "Delirium, subacute" OR "subacute Deliriums") AND TITLE-ABS-KEY ("Stress Disorders, Post-Traumatic" OR "Post-Traumatic Stress Disorder" OR "Stress Disorder, Post-Traumatic" OR "Post Traumatic Stress Disorder" OR "Neuroses, Post-Traumatic" OR "Neuroses, Post Traumatic" OR "Post-Traumatic Neuroses" OR "PTSD" OR "Stress Disorder, Post Traumatic" OR "Post-Traumatic Stress Disorders" OR "Post Traumatic Stress Disorders" OR "Posttraumatic Stress Disorders" OR "Posttraumatic Stress Disorder" OR "Stress Disorder, Posttraumatic" OR "Stress Disorders, Posttraumatic" OR "Neuroses, Posttraumatic" OR "Posttraumatic Neuroses" OR "Acute Post-Traumatic Stress Disorder" OR "Acute Post Traumatic Stress Disorder" OR "Chronic Post-Traumatic Stress Disorder" OR "Chronic Post Traumatic Stress Disorder" OR "Delayed Onset Post-Traumatic Stress Disorder" OR "Delayed Onset Post Traumatic Stress Disorder" OR "Moral Injury" OR "Injury, Moral")
CINAHL	(SU"delirium" OR TI("Delirium of Mixed Origin" OR "Mixed Origin Delirium" OR "Mixed Origin Deliriums" OR "Subacute Delirium" OR "Deliriums, Subacute" OR "Delirium, Subacute" OR "Subacute Deliriums") OR AB("Delirium of Mixed Origin" OR "Mixed Origin Delirium" OR "Mixed Origin Deliriums" OR "Subacute Delirium" OR "Deliriums, Subacute" OR "Delirium, Subacute" OR "Subacute Deliriums")) AND (SU"Stress Disorders, Post-Traumatic" OR TI("Post-Traumatic Stress Disorder" OR "Stress Disorder, Post-Traumatic" OR "Post Traumatic Stress Disorder"OR "Neuroses, Post-Traumatic" OR "Neuroses, Post Traumatic" OR "Post-Traumatic Neuroses" OR "PTSD" OR "Stress Disorder, Post Traumatic" OR "Post-Traumatic Stress Disorders" OR "Post Traumatic Stress Disorders" OR "Posttraumatic Stress Disorders" OR "Posttraumatic Stress Disorder" OR "Stress Disorder, Posttraumatic" OR "Stress Disorders, Posttraumatic" OR "Neuroses, Posttraumatic" OR "Posttraumatic Neuroses" OR "Acute Post-Traumatic Stress Disorder" OR "Acute Post Traumatic Stress Disorder" OR "Chronic Post-Traumatic Stress Disorder" OR "Chronic Post Traumatic Stress Disorder" OR "Delayed Onset Post-Traumatic Stress Disorder" OR "Delayed Onset Post Traumatic Stress Disorder" OR "Moral Injury" OR "Injury, Moral" OR "Moral Injuries") OR AB("Post-Traumatic Stress Disorder" OR "Stress Disorder, Post-Traumatic" OR "Post Traumatic Stress Disorder"OR "Neuroses, Post-Traumatic" OR "Neuroses, Post Traumatic" OR "Post-Traumatic Neuroses" OR "PTSD" OR "Stress Disorder, Post Traumatic" OR "Post-Traumatic Stress Disorders" OR "Post Traumatic

	Stress Disorders" OR "Posttraumatic Stress Disorders" OR "Posttraumatic Stress Disorder" OR "Stress Disorder, Posttraumatic" OR "Stress Disorders, Posttraumatic" OR "Neuroses, Posttraumatic" OR "Posttraumatic Neuroses" OR "Acute Post-Traumatic Stress Disorder" OR "Acute Post Traumatic Stress Disorder" OR "Chronic Post-Traumatic Stress Disorder" OR "Chronic Post Traumatic Stress Disorder" OR "Delayed Onset Post-Traumatic Stress Disorder" OR "Delayed Onset Post Traumatic Stress Disorder" OR "Moral Injury" OR "Injury, Moral" OR "Moral Injuries"))

Table 2 Median-to-mean conversion

Study	Delirium Mean （SD）	Delirium Median (IQR)	Delirium Sample	No Delirium Mean （SD）	No Delirium Median (IQR)	No Delirium Sample
Basinski 2010	0.94 (1.18)		23	0.41 (0.49)		29
Slor 2013	19 (5.3)		23	17.8 (5.3)		30
Svenningsen 2015[a]	-	1.06 (1.00; 1.81)	129	-	1.06 (1.00; 1.63)	118
Bulic 2020	30.3572 (32.0901)	30 (10–51)	27	13.4914 (19.3738)	11 (2–27)	33
Miyamoto 2021	19.81 (24.9167)	22 (4–34)	13	11.0672 (14.7048)	10 （2–21）	34
Su 2024	26.7051 (6.0455)	26 (23–31)	76	-	24 (22–28)	77

Remarks:

a represents median（10; 90 percentile）

- represents non-normal distribution, which cannot be converted.

Figure 1 Forest plot of unadjusted odds ratios (n=8)

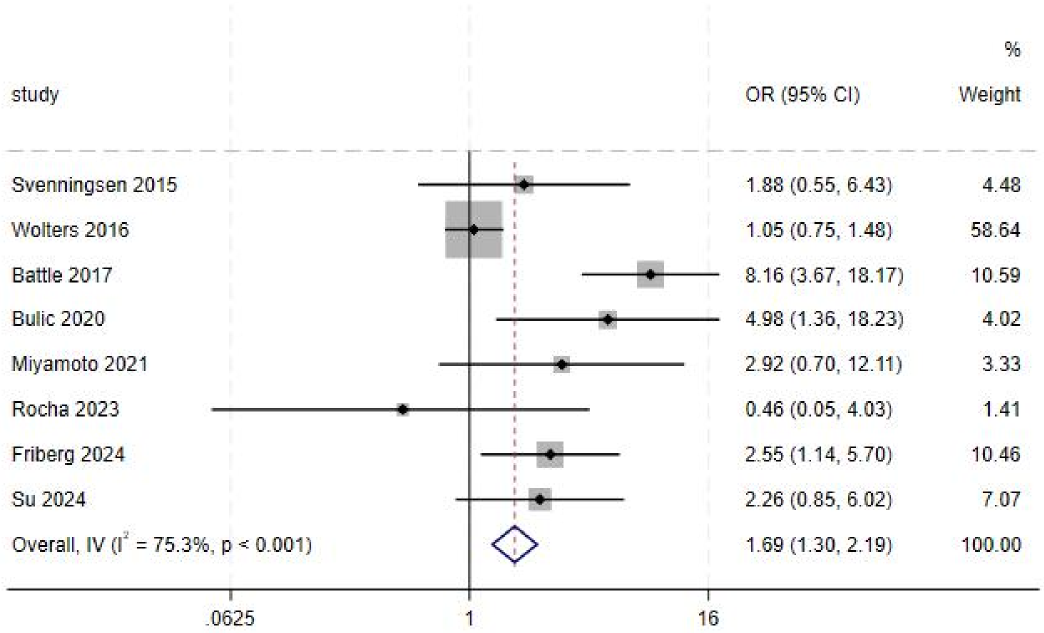

Figure 2 Sensitivity analysis (adjusted odds ratio)

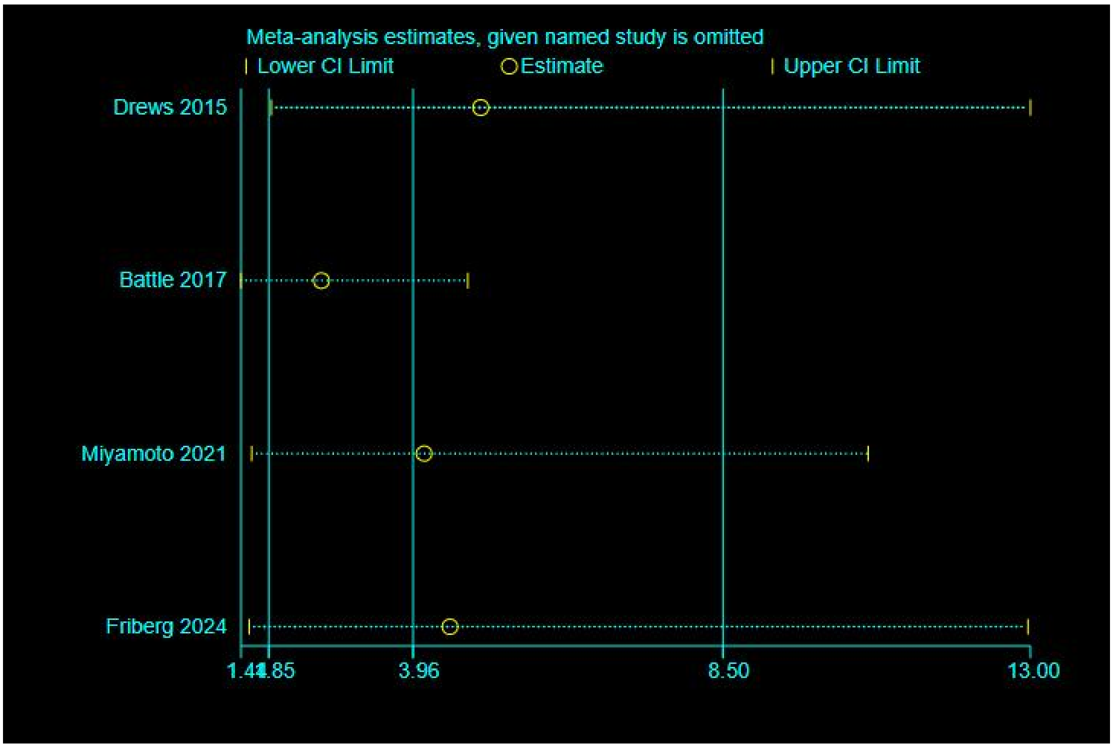

Figure 3 Sensitivity analysis (continuous variables)

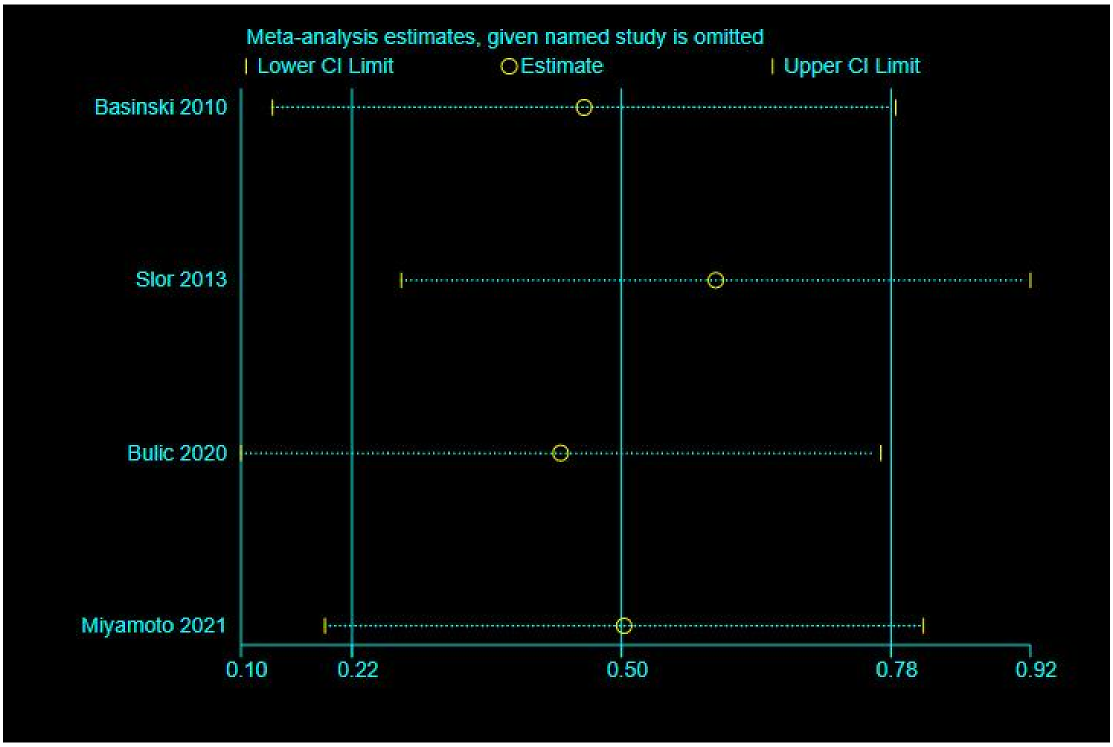

Supplement: Supplementary file 1 [file DataSheet1.pdf]
